# Practical Dissections

**Published:** 1867-05

**Authors:** 


Practical Dissections. By Richard M. Hodges, M.D., formerly

Demonstrator of Anatomy in the Medical Department of Harvard University. Second edition, thoroughly revised. Philadelphia: HENRY C. LEA, 1867. pp. 285.

This is a plain, concise, and convenient guide to the student in his dissections. It is not a substitute for a treatise on anatomy, but just what the student needs for use in the dissecting room. For sale by W. B. KEEN & Co., 148 Lake St.

